# Effect of Microwave Irradiation on Acid Hydrolysis of Faba Bean Starch: Physicochemical Changes of the Starch Granules

**DOI:** 10.3390/molecules27113528

**Published:** 2022-05-31

**Authors:** Mayra Esthela González-Mendoza, Fernando Martínez-Bustos, Eduardo Castaño-Tostado, Silvia Lorena Amaya-Llano

**Affiliations:** 1 Facultad de Química, Universidad Autónoma de Querétaro, Cerro de las Campanas S/N, Querétaro 76010, Mexico; gonzalez.mendoza.mayra@gmail.com (M.E.G.-M.); ecastano@uaq.mx (E.C.-T.); 2 Centro de Investigación y Estudios Avanzados del Instituto Politécnico Nacional, Unidad Querétaro, Libramiento Norponiente 2000, Real de Juriquilla, Querétaro 76230, Mexico; fmartinez@cinvestav.mx

**Keywords:** microwave energy, pasting properties, legume starch, microstructure

## Abstract

Starch is the most abundant carbohydrate in legumes (22–45 g/100 g), with distinctive properties such as high amylose and resistant starch content, longer branch chains of amylopectin, and a C-type pattern arrangement in the granules. The present study concentrated on the investigation of hydrolyzed faba bean starch using acid, assisted by microwave energy, to obtain a possible food-grade coating material. For evaluation, the physicochemical, morphological, pasting, and structural properties were analyzed. Hydrolyzed starches developed by microwave energy in an acid medium had low viscosity, high solubility indexes, diverse amylose contents, resistant starch, and desirable thermal and structural properties to be used as a coating material. The severe conditions (moisture, 40%; pure hydrochloric acid, 4 mL/100 mL; time, 60 s; and power level, 6) of microwave-treated starches resulted in low viscosity values, high amylose content and high solubility, as well as high absorption indexes, and reducing sugars. These hydrolyzed starches have the potential to produce matrices with thermo-protectants to formulate prebiotic/probiotic (symbiotic) combinations and amylose-based inclusion complexes for functional compound delivery. This emergent technology, a dry hydrolysis route, uses much less energy consumption in a shorter reaction time and without effluents to the environment compared to conventional hydrolysis.

## 1. Introduction

Pulses contain a large amount of slow-release carbohydrates, they have a high level of protein (18 to 25 g/100 g) and contain minerals and vitamins. Some of the most important legumes are beans (*Phaseolus vulgaris* L.), lentils (*Lens culinaris* L.), peas (*Pisum sativum* L.), faba beans (*Vicia faba* L.), and chickpeas (*Cicer arietinum* L.). Since they are a high-starch source, their extraction and use are of great interest [1]. Starch is principally composed of two anhydroglucose polymers (amylopectin and amylose) linked by α-(1,4) bonds in linear segments. Additionally, the first has branching points connected to the main chain by an α-(1,6) link. Amylopectin molecules possess a unique structure that confers a substantial degree of crystallinity to the molecules [2]. The crystal structure is divided into three types, A, B, and C, which differ within the compaction limit. In legume starches, type C is the most common and the most resistant to digestion, whereas A is characteristic for cereals [3]. Moreover, a type C crystalline structure implies a mixture of types A and B. Nevertheless, the percentage of each one can vary depending on the source [4] and, consequently, the amount of RS produced during retrogradation. RS refers to the amount of starch and products of starch degradation that have not been absorbed in the gut of healthy humans [5]. Moreover, this fraction passes through the colon, where the microbiota ferments it and mainly produces short-chain fatty acids. Because of this fact, RS has positive effects on diverse diseases [6,7]. Compared to most used starches, pea, lentil, and faba bean starches have relatively high amylose contents, longer branch chains of amylopectin, and the characteristic C-type polymorphous arrangement in the granules. Legume starches also contain a lot of resistant starch (RS) compared to cereal starches [1]. Related to bean starch, faba bean starch has a higher proportion of carbohydrates [8] and differs in its pasting properties [9].

Native starches are modified by diverse methods (physical, chemical, or enzymatic methods) and they are extensively used in the food industry. Starch modified by acid hydrolysis alters the granular structure of starches, resulting in a different behavior upon heating in water and produce pastes with lower intrinsic viscosity values, increased water solubility, and good film-forming properties [10]. These types of starches are used in industry for various applications, for example, thickeners in the food industry or as a wall material to encapsulate diverse food ingredients and active compounds. This latter application is of great interest and mainly modified starches are used. Starches that contain high levels of RS or amylose, have the capability to produce a structural network that facilitates the formation of round-shape microcapsules after spray drying [11]. In addition, starches with high amylose can form amylose-based inclusion complexes of great interest in a wide variety of fields, including drugs, microorganisms, functional compounds delivery, food science, and separation industries. There are currently increasing investigations of RS matrices with protectants to formulate symbiotic (prebiotic/probiotic) combinations for microencapsulation and the delivery of targeted probiotic bacteria into the colon [12].

The functional properties of pulse starches can be modified using emerging technologies such as microwave energy, which has several advantages, including energy-saving, high conversion, rapidity, and no effluents to the environment [13]. Microwave energy accelerates the depolymerization of starch and has been used to modify the physicochemical properties of starch [14,15,16,17,18]. The susceptibility of different starches to microwave irradiation depended not only on their crystalline structure but also on their amylose content [19]. Microwave irradiation creates heat inside the processed materials because of rapid alterations of the electromagnetic field at high frequencies [20]. Hence, microwave irradiation has an effect in a shorter process time, with higher yield, and better quality of products than those obtained by conventional processing techniques [21]. Using microwave irradiation, the starch was completely hydrolyzed within 5 min in a suspension of starch (10 g/100 mL) in hydrochloric acid (0.5 M) [22]. Warrand et al. [23] hydrolyzed pure amylose under acidic conditions by microwave irradiation and conventional heating. Microwave was shown to be more efficient than conventional heating. In addition, acid microwave heating directly transformed starch and fiber into depolymerized products and resulted in simpler sugars than autoclaving [24]. These investigations showed that the presence of acid can enhance the effects of microwave energy, while coupling accelerates the hydrolysis.

Faba bean, due to its features mentioned before, has the potential to be used as a food-grade material. Thus, the focus of the current investigation was to prepare hydrolyzed faba bean starch using acid assisted by microwave energy. Hydrolysis using microwave energy is an environmentally friendly process because it has low energy consumption, a shorter reaction time, and no generation of effluents.

## 2. Results and Discussion

### 2.1. Starch Isolation

The yield of starch extraction from faba bean seeds (*Vicia faba* var. major) was 30–33 g/100 g, similar to the one reported by Ambigaipalan et al. [25]. These authors reported mean values of 34.6 of pulse starches isolated from different cultivars of faba bean. Due to the existence of insoluble proteins, fiber, and minerals within starch granules, it is difficult to obtain pure legume starch [26]. The purity of starch is influenced by the extraction method. In this work, the wet method was used, which has a lower yield. However, starches with a higher degree of purity are achieved [27]. NFBS showed 8.3 ± 0.14% moisture content, within the range (7–15%) obtained for native legume starches [28]. The ash content was 0.07 ± 0.029 g/100 g, similar to that reported by Zhang et al. [26] and Piecyk and Domian [1] for faba bean seed. Regarding the protein content, the value (0.85 ± 0.012 g/100 g) was higher than the one reported by Ambigaipalan et al. [25] and Zhang et al. [26], which was 0.38 and 0.30 g/100 g, respectively, for faba bean starch. Other authors, such as Hoover et al. [28], reported values similar to those obtained in this work for the same legume. These differences may be due to the extraction process used. Concerning the lipid content, this was 2.0 ± 0.03 g/100 g, being higher than the one reported by Hoover et al. [28]. The purity of starch can be qualified by the composition of ash and protein [27]. Thus, the results indicate that the starch obtained has a high degree of purity.

### 2.2. Characterization of Native and Hydrolyzed Faba Bean Starches

**Figure 1:**
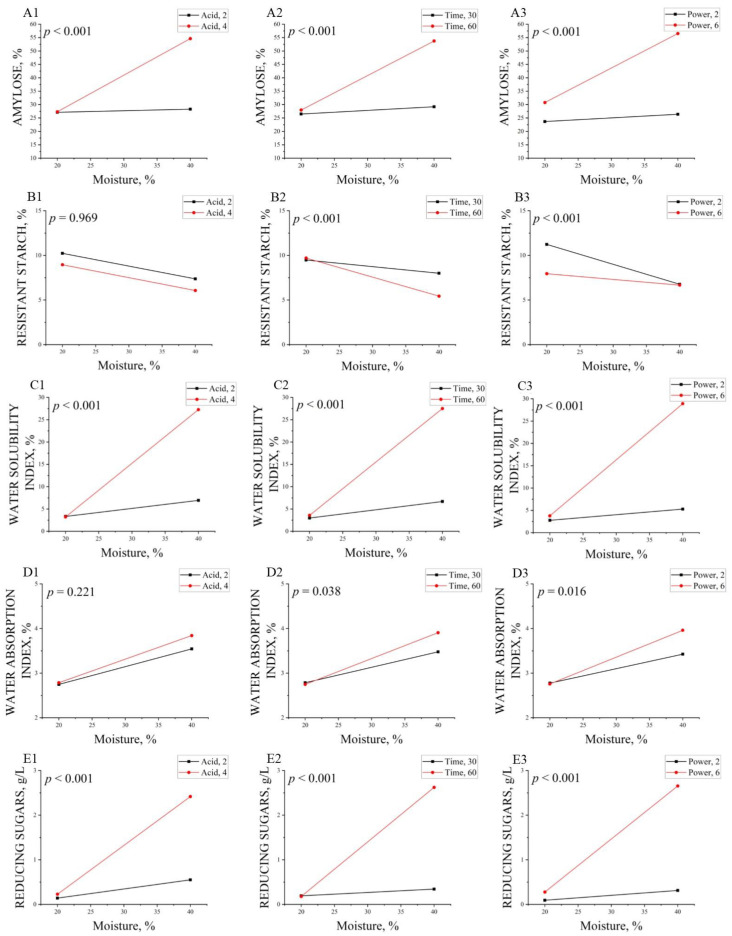
Interaction effects of moisture with pure hydrochloric acid, moisture with time, and moisture with power level. (**A1**–**A3**), interactions effects for amylose; (**B1**–**B3**), interactions effects for resistant starch; (**C1**–**C3**), interactions effects for water solubility index; (**D1**–**D3**), interactions effects for water absorption index; (**E1**–**E3**), interactions effects for reducing sugars.

The characterization of native faba bean starch (NFBS), conventional hydrolysis faba bean starch (CFBS), and hydrolyzed faba bean starches (HFBS) is presented in Table 1. Results for NFBS agreed with those previously reported [1,26,27]. Amylose and RS contents showed values of 38.3 and 12.8 g/100 g, respectively. Lower values (from 25.8 to 33.6 g/100 g) were reported for amylose and higher for RS (from 8.1 to 15.0 g/100 g) for pulse starches isolated from different cultivars of faba bean [27]. All the assays processed with microwave energy (MWE) and hydrochloric acid (HA), including CFBS, showed lower values of amylose and RS than native faba bean starch. The amylose content that resulted after the hydrolysis of starch varied according to the starch source and hydrolysis conditions. Existing knowledge on microwave treatment of starches with completely different amylose contents suggest that the MWE is preferably transmitted to the amorphous region of the starch granule, and the crystalline regions are affected afterwards [10,29]. The hydrolysis conditions of faba bean starch slightly decreased the liberation of amylose, although the application of severe conditions of moisture (M), HA, and MWE (HFBS8 treatment) resulted in the highest amylose content and the lowest RS. The acid hydrolysis decreased viscosity and increased solubility. In addition, when the reaction time of the hydrolysis of maize starch was increased, the crystallinity of the starch increased while its amylose content decreased [30]. Microwave treatment reduced the degree of amylopectin branching on lotus seed starch leading to the degradation of linear chains and the reduction of crystal regions, which further promoted the formation of RS during cooling [21]. Amaya-Llano et al. [31] reported that HA concentration from 1–3 mL/100 mL reduced amylose of jicama and maize starches, nevertheless a great increase (3–5) raised the amylose content of both starches. This increase of amylose was attributed to the fast degree of depolymerization of amylopectin. Aaliya et al. [32] reported that use of MWE showed a significant difference in the swelling and solubility of talipot starches. It can be seen that there are no significant differences between NFBS and CFBS in solubility and absorption indexes, similar to that reported by Cruz-Benítez et al. [33] for cassava starch. HFBS8 showed major differences due to the clear depolymerization of the granules. Therefore, the water solubility index (WSI) increased from 2.5 to 49.5%, which can also be corroborated by the rise in reducing sugars up to 4.7 g/L. Such behavior was similar to that reported by González et al. [34] in lentil starch modified by microwave irradiation. The HFBS8 treatment was able to retain water inside its structure, maybe because of the recrystallization process. However, another possibility could be that this intense treatment caused structural changes in the amylopectin molecules, resulting in the formation of longer amylopectin chains due to fusion of the granules and exposed more hydroxyl groups, thereby increasing the swelling power [35]. To understand the mechanism of the microwave acid hydrolysis phenomenon, two-way interaction effects on each response variable were interpreted. All two-way interactions effects were statistically significant for each response variable, except for RS and the water absorption index (WAI) in the interaction M with pure hydrochloric acid (A) (Figure 1(B1,D1)). The interactions effects on amylose concentration (Figure 1(A1–A3)), WSI (Figure 1(C1–C3)) and reducing sugars (Figure 1(E1–E3)) show that only having low acid concentration, short time, and low power level (P), when the moisture is modified from 20 to 40%, does not produce a statistical change. The explanation of the behavior could be that at these levels, the molecules do not reach a sufficient state of excitation to modify the starch structure to a great extent [14]. While for high levels of each factor, it was observed that hydrolysis occurs and, therefore, an increase in amylose, solubility and reducing sugars. For the effects of the significant interactions on RS (Figure 1(B2,B3)), it can be observed that M affects RS only when Time = 60 or Power = 2. It has been reported that RS content decreases depending on the degree of acid hydrolysis and its conditions, namely, temperature, time, type of acid, and concentration [7,36,37]. The non-significant interaction between M and A (Figure 1(B1)) shows that RS decreases when M augments. This reduction is independent of the value of A, probably because both factors can modify starch. M improves the molecular movement in MWE conditions, cleaving amylopectin and amylose chains [17,18], while A can itself modify starch by the action of the hydrogen ions that react with the oxygen atom of α- (1,4) or α-(1,6) glycosidic bond [38]. Therefore, both factors additively decreased the RS content. High levels produced materials with less concentration of RS, maybe due to severe hydrolysis. Concerning significant interaction effects on WAI (Figure 1(D2,D3)), it can be observed that when moisture content increases from 20 to 40%, the water absorption will always increase, especially when Time = 60 or Power = 6. The non-significant two-way interaction effect, corresponding to M with A (Figure 1(D1)), shows that WAI rises when M increases. This rise is independent of the value of A, probably due to the rupture of starch granules caused by the modification of each factor, thus resulting in the exposure of more hydroxyl groups that can form hydrogen bonds with water similar to that observed by Li et al. [15]. Starches may present a two-step hydrolysis scheme: a rapid initial velocity followed by a later slower velocity. The relatively fast initial rate corresponds to the hydrolysis of the amorphous zones, whereas the slow process is attributed to the simultaneous hydrolysis of the amorphous and crystalline regions. The first hydrolysis stage is influenced by the size of the granules, the pores on the surface, the amylose content, and the amount of lipid-complexed amylose chains. The second hydrolysis step is influenced by the amylopectin content, the distribution of α-(1,6) branches between the amorphous and crystalline lamellae, and the degree of packing of the double helices within the crystallites [10]. The results suggest that with low levels in each factor the hydrolysis of the amorphous parts commence, while with high levels, hydrolysis of the amorphous and crystalline regions may occur. A similar effect was reported for hydrolyzed potato starch using induced electric field and HCl [38].

### 2.3. Determination of Pasting Properties

**Figure 2:**
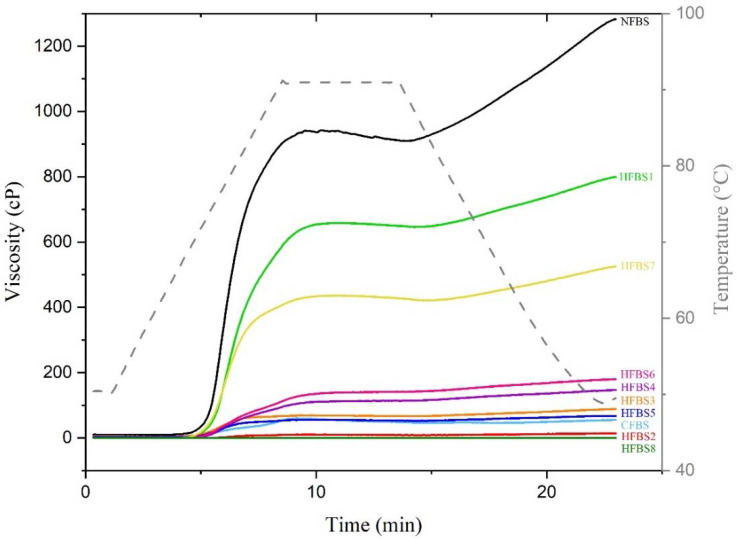
Brabender viscosity curves of native and hydrolyzed faba bean starches. NFBS, native faba bean starch; CFBS, conventional hydrolysis faba bean starch; HFBS1, HFBS2, HFBS3, HFBS4, HFBS5, HFBS6, HFBS7, and HFBS8, hydrolyzed faba bean starches.

The viscosity profiles of NFBS, CFBS, and HFBS are shown in Figure 2 and the pasting parameters in Table 2. Results for NFBS were similar to those previously reported by Ambigaipalan et al. [25] for faba bean starch. The viscosity parameters of CFBS and HFBS with diverse M, HA, and MWE conditions statistically decreased regarding NFBS. The lowest viscosity values were for the starches with the combination of more severe HA and MWE conditions. The use of MWE in acidic conditions allowed the production of starches with different characteristics than other starches treated with MWE [14,15,17,18]. According to Luo et al. [39] the viscosity patterns of waxy and normal starches remained unchanged, while amylomaize V starches changed on microwave treatment. After microwave irradiation, an increase in pasting temperature and a drop in viscosity of the three starches were observed. In a microwave field, polar compounds like water molecules and HCl, vibrate at very high frequency, and then the rapid friction, collision, and vibration between water molecules and starch granules generate heat during a short period, which can cause the physical damage of the starch granules and the degradation of structures in a shorter time [15]. Materials used as encapsulants for food should have some characteristics, including biocompatibility, water solubility, emulsifying and film forming properties, and low viscosity at high concentrations. Additionally, the use of starches with other desirable characteristics includes high amylose and RS [40]. MWE changed the viscosity properties of waxy and non-waxy rice starches containing 20% moisture [41]. In our work, all the starches processed with M, HA, and MWE, decreased in their pasting properties, indicating that the severity of the combined processing parameters resulted in low re-aggregation of starch granules. The breakdown viscosity values also decreased, and HFBS8 showed undetectable parameters of viscosity by RVA. The optimal response variables for potential use as wall material for the encapsulation could be starches with the highest WSI, the lowest final viscosity, and starches with different amylose content. Starch with high amylose content can exhibit a low release behavior, and starch with high amylopectin content can offer high core material stability and protection [42]. Thus, for the subsequent analysis, HFBS2, HFBS5, and HFBS8 treatments can be potentially considered as possible food-grade coating material.

### 2.4. Thermal Properties by Differential Scanning Calorimetry (DSC)

In Table 3, the parameters of NFBS, CFBS, and HFBS are presented. Results for NFBS for the same legume were similar to those previously reported by Ambigaipalan et al. [25], Li et al. [27], and Piecyk et al. [1]. The gelatinization temperatures increased on the hydrolyzed starches (CFBS, HFBS2, HFBS5, and HFBS8) in comparison to NFBS. The microwave melted off weak crystallites and formed strong ones, increasing the T_o_, T_p_, and T_f_ [15]. This shift is considered to reflect an increase in gelatinization temperature due to the increased molecular order in acid hydrolyzed starch. Further interpretations include the preferential hydrolysis of amorphous regions that attenuate the destabilizing effect of swelling in amorphous regions on the melting of the crystallites, or longer amylopectin double helices may be formed as a result of the removal of branch points [10]. In addition, the gelatinization enthalpy (ΔH) of CFBS was not significantly different from NFBS, similar to the one reported by Atichokudomchai et al. [43] in tapioca starch, hydrolyzed by HCl at room temperature for 192 h. It is suggested that the partially hydrolyzed amylose retrograded, leveling the double helix content (which otherwise would decrease with hydrolysis), so that ΔH did not decrease. Even in the presence of a certain hydrolysis of the crystalline domain, the enthalpy of fusion did not diminish. The ΔH of the samples HFBS2 and HFBS8 was decreased by 76.4% and 94.5%, respectively. ΔH reflected mainly the loss of the double-helix and the molecular order in the crystalline region. The lower ΔH suggested the lack of order of the crystalline region and decreased stability of the crystalline and amorphous regions, while the energy required for further destruction of the crystalline regions was diminished [15]. Therefore, the results suggest that the conditions of moisture content (40%) and power level (6) in HFBS2 and HFBS8 were sufficient to lose the molecular order of the starch. The difference in enthalpy between them is due to the acid concentration and time of exposure to MWE. In the HFBS2 treatment, the levels of these factors were 2 mL/100 mL of HA and 30 s, while for HFBS8, they were 4 mL/100 mL and 60 s, respectively. The polar molecules and ions vibrate with the application of MWE, generating thermal energy [44]. Hence, as there were more polar molecules in the medium and the time was longer, the temperature increased as well as the modification of the starch (HFBS8 > HFBS2). Finally, for HFBS5, no statistical changes were observed because the moisture (20%) of the treatment was not enough to modify the molecular order in the crystalline region, similar to that reported by Li et al. [15].

### 2.5. X-ray Diffraction

**Figure 3:**
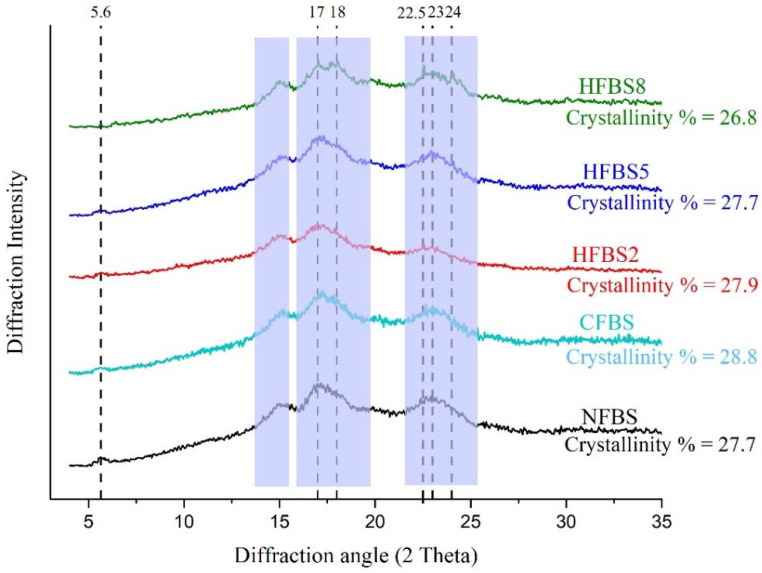
XRD patterns and relative crystallinity of native and hydrolyzed faba bean starches. Regions highlighted in color are the most affected peaks. NFBS, native faba bean starch; CFBS, conventional hydrolysis faba bean starch; HFBS2, HFBS5, and HFBS8, hydrolyzed faba bean starches.

The XRD spectra of NFBS, CFBS, and HFBS are shown in Figure 3. NFBS exhibited the characteristic “C-type” XRD patterns, which agreed with previous reports [1,25,26] for the same legume. The acid does not modify the crystalline characteristics of A- and B-type starches and is commonly used to investigate the allomorph distribution of C-type starch [45]. The XRD spectra of acid hydrolysis starches (CFBS, HFBS2, and HFBS5) showed that the peak at 5.6° 2θ was reduced in intensity and the peak at 17°, 18°, and 23° 2θ sharpened (Figure 3), similar to the one reported in faba bean starch hydrolyzed by α-amylase-HCl [9]. Hydrolyzed potato starch using induced electric field-HCl treatment did not cause variation of the crystalline type [38]. Nevertheless, HFBS8 showed that the peak at 5.6° 2θ appeared with weak intensity and the peak at 17°, 18°, 22.5°, and 24° 2θ sharpened (Figure 3). These results showed a pattern of B-type starch, which agrees with the results of Polesi et al. [46] for retrograded chickpea starch. Retrogradation at low temperatures leads to the formation of B-type crystallinity. As mentioned earlier, in HFBS8, the moisture and temperature conditions were suitable for the gelatinization of the starch and its subsequent recrystallization. The transition from C to A or C to B could be predominantly attributed to the preferential hydrolysis of one polymorph followed by the possible rearrangement of decoupled double helices [10]. The relative crystallinity (RC) of NFBS was 27.7% and hydrolyzed starches were in the range of 26.8–28.8% (Figure 3). Ambigaipalan et al. [25] have shown that native faba bean starch has a 20.2–21.9% of RC, lower than our results. However, the values are within the range (17.0–34.0%) reported for other pulse starches [28]. RC in CFBS increases a little (From 27.7 to 28.8%). The difference in RC could be attributed to the size of the crystal and the orientation of the double helix in the crystal domain [47]. There was a significant correlation between the degree of retrogradation and crystallinity, attributed to the development of a more ordered or crystalline state formed during the retrogradation [48]. When starch granules are submitted to acidic hydrolysis, the RC increases with the time of hydrolysis. Several hypotheses have been suggested for the enhanced crystallinity in the initial stages of acid hydrolysis. First, the cleavage of some of the amylose chains running through the amorphous regions may allow reordering of the newly released chain ends into a more crystalline structure. Second, the reordering of the crystalline structure during acid hydrolysis would result in increased crystallinity by partial filling of water channels in the crystallite cavities with double helices. Third, increased crystallinity may also result from the retrogradation of hydrolyzed free amylose into double helices, which rearrange into crystalline regions that are resistant to acid hydrolysis [10]. The lowest RC was 26.8% for HFBS8, probably due to the more intense rupture of the gelatinized starch through the action of acid. Similar results were reported for chickpea starch, retrograded and gelatinized by acid hydrolysis [46]. Progressive loss of starch crystallinity upon increased duration of the microwave treatment was also reported by Kumar et al. [14], caused by the capacity of microwaves to directly damage the lamellar arrangement of amylopectin crystals by resonating water molecules, and in this work also by HCl molecules.

### 2.6. Fourier Transform-Infrared Spectroscopy

**Figure 4:**
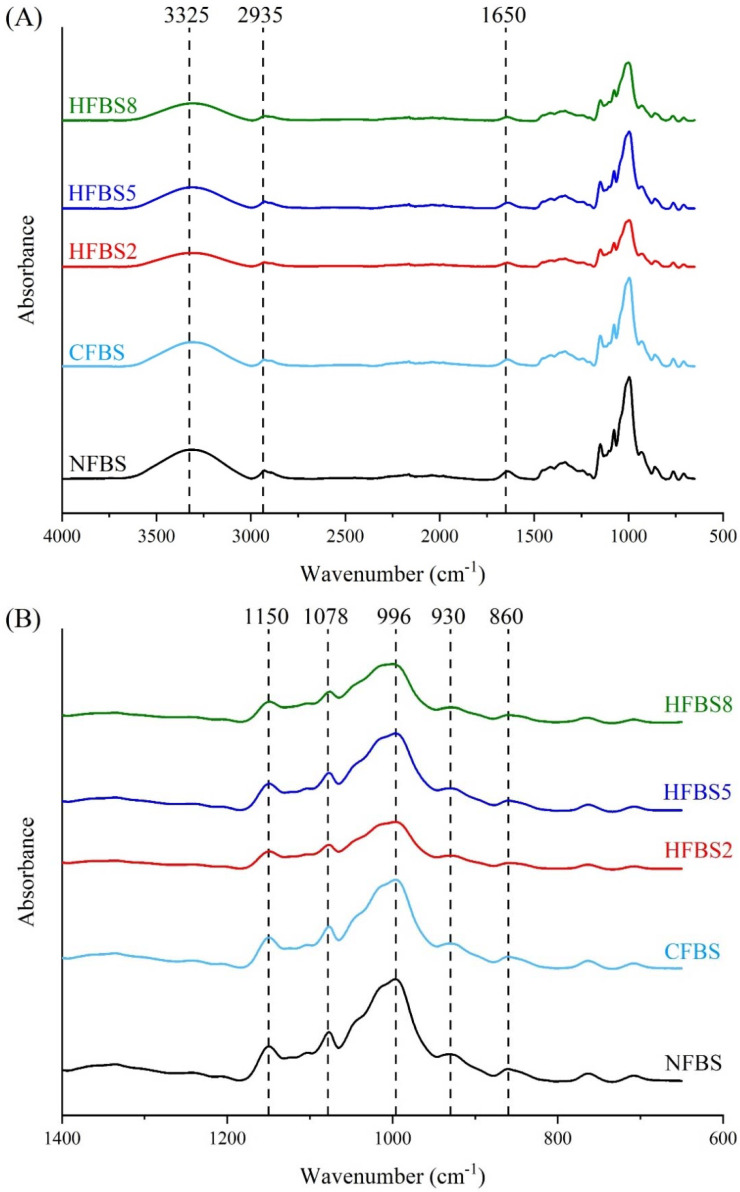
FTIR spectra. (**A**) Patterns of native and hydrolyzed faba bean starches. (**B**) The fingerprint characteristic peaks. NFBS, native faba bean starch; CFBS, conventional hydrolysis faba bean starch; HFBS2, HFBS5, and HFBS8, hydrolyzed faba bean starches.

The FTIR spectra of NFBS, CFBS, and HFBS are shown in Figure 4A. Broad peaks appeared approximately at 3325 cm^−1^, indicating the presence of hydroxyl groups (O–H). Peaks at 2935 cm^−1^ and 1650 cm^−1^ representing the C–H stretching vibration and bound water present in the starch, respectively, were also seen in the FTIR spectra. The fingerprint region of the starch spectrum (Figure 4B) has five characteristic peaks between 800 and 1200 cm^−1^, attributed to C–O bond stretching. The peak around 996 cm^−1^ is attributed to the C–O of the C–O–C in the polysaccharide; the peaks close to 1078 and 1150 cm^−1^ are characteristic of the anhydroglucose ring C–O stretch; the peak near to 930 cm^−1^ was assigned to the skeletal mode vibration of α-(1–4) glycosidic linkage, while the peak near to 860 cm^−1^ corresponds to C–H and CH_2_ deformations [49]. There was neither the appearance of a new peak nor loss of a peak, suggesting no new formation or loss of chemical bonds, which indicated that microwave treatment did not change the starch molecules, as reported by Li et al. [15].

### 2.7. Morphology of Starch Granules

**Figure 5:**
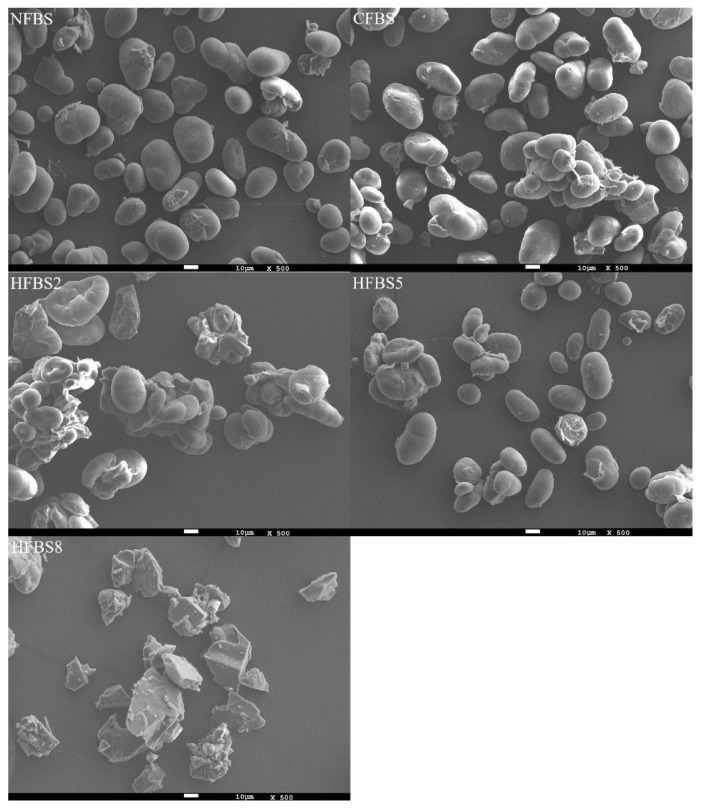
SEM images of native and hydrolyzed faba bean starch. The magnification of images was 500x. NFBS, native faba bean starch; CFBS, conventional hydrolysis faba bean starch; HFBS2, HFBS5, and HFBS8, hydrolyzed faba bean starches.

SEM micrographs of NFBS, CFBS, and HFBS appear in Figure 5. Granules of NFBS were round, elliptical, and oval shaped, similar to that reported by [1] for the same legume. There were some damaged granules. This could be due to the extraction method used, which can damage the granules because of the dry and wet grinding. However, damaged granules were rarely observed. The granule size of NFBS was of length, 28.2 ± 6.68 µm and width, 17.7 ± 3.92 µm. These findings were consistent with Zhang et al. [26] for native faba bean starch. The starch granule structure of CFBS and HFBS5 was not completely destroyed, and the original aspect of the granules was maintained but presented some agglomerations, characteristic of starches that have been hydrolyzed. The starch granules with a low degree of hydrolysis remained intact, although the outer surface became roughened and could present some agglomerations [10]. Starch systems with low moisture content (<30%) have poor absorption and conversion abilities, allowing the morphology of starch granules to remain completely intact with controlled microwave treatment [44], and this was observed in HFBS5 with 20% moisture. The rough surface and agglomerations observed could be due to the effect of acid in the medium or be associated with the presence of water in the granules with the combination of MWE [29]. The granule sizes of CFBS and HFBS5 were of lengths, 30.2 ± 10.36 and 27.0 ± 9.35 µm, and widths, 19.4 ± 6.38 and 17.9 ± 8.41 µm, respectively. For HFBS2, the surface became roughened with wide cracks and presented agglomerations to form larger starch clusters. The granule size was of length, 63.5 ± 20.65 µm and width, 43.5 ± 13.75 µm. This damage could be explained by the high internal steam pressure formed by the rapidly heating water, which also suggests that the internal structure of the starch disintegrated [15]. It appears that the starch granules were merged with the adjacent granules after the microwave treatment. For HFBS8, the round, elliptical and oval shapes of the starch disappeared entirely, and the large blocks formed, with a length of 40.5 ± 14.82 µm and width, 24.8 ± 7.53 µm. This indicated that when the moisture content was 40%, the excessive water and high temperature could gelatinize the starch, similar to that reported by Li et al. [15]. This treatment also had a high acid concentration and the longest MWE time, contributing to the rapid hydrolysis of the starch and gelatinization.

## 3. Materials and Methods

### 3.1. Materials

Faba bean seeds (*Vicia faba* var. major) were acquired in a local market of Queretaro, Queretaro, Mexico (Latitude: 20°35′17″ N; longitude: 100°23′17″ W). All reagents were analytical grade.

### 3.2. Starch Isolation

Starch was isolated from faba bean seeds according to Li et al. [15] with minor modifications. Seeds were ground in a granulator mill with a blade system and an integrated mesh to obtain a fine powder (flour). The flour (~300 g) was suspended in 750 mL of distilled water and stirred for 1 h. After the supernatant was carefully decanted, the protein layer on top of the precipitate was removed. This step was repeated twice. The pellet was resuspended in 600 mL of distilled water, followed by adding 2 g/100 mL NaOH and stirring for 1 h. The suspension was allowed to stand 12 h at room temperature. Subsequently, a wet grinding was carried out with a stone mill (FUMASA). The suspension obtained was submitted to sieving analysis, with the following set of Tyler sieves: 30, 100, 200 mesh, and a pan (U.S.). The residues were washed with water. The starch was left to stand overnight at 5 °C with 0.2 g/100 mL sodium bisulfite. The supernatant was removed, and the residue was dried at 40 °C in a convection oven for 24 h. The dried starch was ground and sieved in a mesh of 250 µm opening size. The NFBS was placed in polyethylene bags and stored at room temperature. NFBS was analyzed for moisture, protein, fat, and ash contents as described by the AOAC [50] methods 925.10, 920.87, 920.85, and 923.03, respectively.

### 3.3. Starch Hydrolysis

#### 3.3.1. Conventional Acid Hydrolysis

Starch was hydrolyzed with hydrochloric acid according to the methodology described by Murúa-Pagola et al. [51]. The dried starch was ground and sieved in a mesh of 250 µm opening size. The CFBS was placed in polyethylene bags and stored at room temperature.

#### 3.3.2. Starch Acid Hydrolysis by Microwave Treatment

A conventional microwave oven (Panasonic NN-SB646S, Beijing, China) was used, the working frequency was 2450 MHz, and the maximum electric power was 1100 W. A fractional factorial design (2^4−1^) for the acid hydrolysis was evaluated. Factors and levels were as follows: moisture, 20% and 40%; pure hydrochloric acid concentration, 2 and 4 mL/100 mL; time, 30 and 60 s; and power level, 2 and 6. The hydrolysis of the starch was carried out as follows. The NFBS was conditioned to moisture content and concentration of HCl according to the design matrix (Table 4). Later, it was stabilized for 12 h at 4 °C. The starch samples were subjected to microwave treatments according to Table 4. After treatment, starch samples were allowed to cool down, and their pH was adjusted to 7.0 with 2 g/100 mL NaOH. Then, they were dried at 40 °C for 24 h, processed in a mill (Krups GX4100, México City, Mexico), and sieved with 60 mesh to obtain the HFBS.

### 3.4. Characterization of Starches

Samples of NFBS, CFBS, and HFBS were analyzed. Enzyme kits determined amylose and RS according to the specifications (Megazyme K-RSTAR and K-AMYL). WAI and WSI were determined using the method described by Anderson et al. [52]. Reducing sugar content was measured by the 3,5-dinitrosalysilic acid method [53].

### 3.5. Determination of Viscosity Properties (RVA)

The viscosity profile was evaluated using a Rapid Visco Analyzer (RVA) model Super 4 (Newport Scientific PTY Ltd., Sydney, Australia). Sample amount of 2 g (dry base) was weighed out, and 28 mL of distilled water was added. Measurements were made according to the specifications of the AACC [54], 61-02 method. The results obtained from the equipment were expressed in units of viscosity (cP).

### 3.6. Thermal Properties by Differential Scanning Calorimetry

Thermal properties were performed using a differential scanning calorimeter (DSC Mettler Toledo, model 821). Sample amount of 3 mg was weighed out, and 7 mg of distilled water was placed in a 40 µL aluminum crucible at room temperature (25 °C), subsequently sealed and allowed to stand for 12 h at room temperature for even distribution of water. The sample was subjected to a heating ramp from 30 to 100 °C, with a 10 °C/min rate. An empty aluminum crucible was used as a reference. The start temperature, the maximum temperature, the completion temperature, and the enthalpy of gelatinization (ΔH) were recorded [15].

### 3.7. X-ray Diffraction

The X-ray diffraction was recorded using an X-ray diffractometer (Rigaku X-ray diffractometer DMAX-2100). Operating conditions included a CuKα radiation of λ = 1.5405, 30 kV, and an electric current of 16 mA. Approximately 1 g of sample was loaded onto a glass plate and scanned in the range of 5° to 60° Bragg angles in steps of 1°/0.03 s at room temperature. The relative crystallinity was determined from the ratio of the areas of the diffraction peaks to the area of the whole diffraction pattern subtracting amorphous background patterns using OriginPro 2018 Software (OriginLab Corp., Northampton, MA, USA) [15].

### 3.8. Fourier Transform-Infrared Spectroscopy

FTIR spectra were produced with a Spectrum GX spectrometer (Perkin Elmer, Waltham, MA, USA) with a diffuse reflectance accessory (Pike Technology model). Samples were prepared by finely grinding starch with KBr in a ratio of 1:100 (*w*/*w*) and scanned over a wavenumber range from 400 to 4000 cm^−1^ [51].

### 3.9. Scanning Electron Microscopy

Morphologies of samples were studied and analyzed by a field emission scanning electron microscopy (JXA-8530F, JEOL, Tokyo, Japan). A drop of suspension was mounted on a silicon wafer and allowed to dry on a desiccator. Subsequently, the wafer was mounted on specimen stubs with carbon black tape and sputter-coated (Denton Vacuum Desk V sputter) with gold and an exposure time of 60 s before observation [51].

### 3.10. Statistical Analysis

All experiments were conducted with three replications. Mean values and standard deviations (SD) were computed. The experimental data were analyzed using analysis of variance (ANOVA). All analysis was performed using R software (version 3.6.1, Vienna, Austria).

## 4. Conclusions

Microwave energy reduced the time required to obtain HFBS from hours to seconds. Microwave treatments combined with acid decreased the amylose content from 38.3 to 22.8 g/100 g (except for HFBS8), while RS, water solubility, and reducing sugars were increased. Furthermore, MWE modified the viscosity profile of starch granules, compared with NFBS and CFBS; the viscosity values statistically decreased. The most severe conditions resulted in the lowest viscosity values, the highest amylose content, solubility, absorption indexes, and reducing sugars. The starch granule structure of samples CFBS and HFBS5 was not totally destroyed, and the original appearance of the granules was maintained but presented some agglomerations. In HFBS8, the original shape of the starch disappeared completely. In summary, we report a broadly applicable and rapid microwave treatment protocol for the development of HFBS with high amylose and low viscosity contents that can be applied as an alternative encapsulant material.

## Figures and Tables

**Table 1:** Physicochemical characterization of native and hydrolyzed faba bean starches ^1^.

Sample	Amylose, g/100 g	RS, g/100 g	Water Solubility Index, %	Water Absorption Index, %	Reducing Sugars, g/L
NFBS	38.3 ± 2.63 ^b^	12.8 ± 0.57 ^a^	1.9 ± 0.36 ^f^	2.5 ± 0.22 ^d^	0.03 ± 0.020 ^e^
CFBS	34.6 ± 0.68 ^bc^	10.6 ± 1.21 ^ab^	2.1 ± 0.09 ^f^	2.7 ± 0.05 ^cd^	0.09 ± 0.010 ^de^
HFBS1	22.8 ± 3.04 ^f^	11.8 ± 0.40 ^a^	2.5 ± 0.13 ^ef^	2.8 ± 0.16 ^cd^	0.06 ± 0.025 ^e^
HFBS2	31.1 ± 4.16 ^cd^	8.6 ± 0.17 ^bc^	8.3 ± 0.29 ^b^	3.6 ± 0.48 ^b^	0.58 ± 0.066 ^b^
HFBS3	30.1 ± 1.99 ^cde^	7.5 ± 1.27 ^cd^	3.4 ± 0.75 ^def^	2.8 ± 0.13 ^cd^	0.33 ± 0.144 ^cd^
HFBS4	27.3 ± 1.56 ^def^	7.4 ± 0.70 ^cd^	5.0 ± 0.52 ^cd^	3.4 ± 0.15 ^bc^	0.11 ± 0.031 ^de^
HFBS5	31.4 ± 1.04 ^cd^	8.7 ± 0.40 ^bc^	4.1 ± 0.82 ^cde^	2.7 ± 0.13 ^cd^	0.23 ± 0.060 ^de^
HFBS6	25.5 ± 0.70 ^def^	6.1 ± 0.06 ^de^	5.5 ± 0.58 ^c^	3.5 ± 0.37 ^b^	0.52 ± 0.113 ^bc^
HFBS7	24.5 ± 1.66 ^ef^	11.0 ± 1.44 ^a^	3.0 ± 0.98 ^ef^	2.8 ± 0.17 ^cd^	0.13 ± 0.020 ^de^
HFBS8	82.0 ± 2.05 ^a^	4.7 ± 0.31 ^e^	49.5 ± 0.93 ^a^	4.3 ± 0.18 ^a^	4.73 ± 0.147 ^a^

^1^ RS, resistant starch; NFBS, native faba bean starch; CFBS, conventional hydrolysis faba bean starch; HFBS1, HFBS2, HFBS3, HFBS4, HFBS5, HFBS6, HFBS7, and HFBS8, hydrolyzed faba bean starches. Assays were performed in triplicate. Mean ± SD, values in the same column with different superscript letters are significantly different (α = 0.05).

**Table 2:** Pasting parameters of native and hydrolyzed faba bean starches ^1^.

Sample	T_ps_ (°C)	η_pk_ (cP)	Δη_bd_ (cP)	Δη_sb_ (cP)	η_f_ (cP)
NFBS	72.5 ± 0.69 ^ab^	975.7 ± 24.58 ^a^	35.3 ± 5.51 ^a^	401.0 ± 15.62 ^a^	1341.3 ± 44.24 ^a^
CFBS	70.2 ± 2.84 ^b^	53.3 ± 40.72 ^ef^	8.7 ± 10.01 ^b^	7.7 ± 1.53 ^de^	52.3 ± 32.01 ^fg^
HFBS1	70.8 ± 1.27 ^b^	655.3 ± 30.55 ^b^	6.3 ± 5.77 ^b^	149.3 ± 15.04 ^b^	798.3 ± 17.79 ^b^
HFBS2	75.1 ± 0.37 ^a^	11.3 ± 8.08 ^f^	2.0 ± 1.73 ^b^	4.7 ± 1.15 ^de^	14.0 ± 9.64 ^fg^
HFBS3	69.8 ± 2.34 ^b^	68.3 ± 11.02 ^def^	1.7 ± 1.15 ^b^	21.7 ± 7.02 ^de^	88.3 ± 17.93 ^def^
HFBS4	70.7 ± 1.61 ^b^	112.0 ± 27.78 ^de^	1.0 ± 0.05 ^b^	35.7 ± 3.06 ^d^	146.7 ± 38.55 ^de^
HFBS5	70.6 ± 1.07 ^b^	54.0 ± 13.23 ^ef^	1.7 ± 1.15 ^b^	15.3 ± 10.78 ^de^	67.7 ± 16.07 ^efg^
HFBS6	70.2 ± 0.43 ^b^	143.3 ± 49.89 ^d^	2.3 ± 1.53 ^b^	31.0 ± 10.53 ^de^	172.0 ± 41.62 ^d^
HFBS7	68.9 ± 1.22 ^b^	446.3 ± 19.55 ^c^	11.7 ± 6.66 ^b^	102.7 ± 23.69 ^c^	537.3 ± 41.48 ^c^
HFBS8	0.0 ± 0.00 ^c^	0.0 ± 0.00 ^f^	0.0 ± 0.00 ^b^	0.0 ± 0.00 ^e^	0.0 ± 0.00 ^g^

^1^ T_ps_, pasting temperature; η_pk_, peak viscosity; Δη_bd_, breakdown viscosity; Δη_sb_, setback viscosity; η_f_, viscosity at end of final holding. NFBS, native faba bean starch; CFBS, conventional hydrolysis faba bean starch; HFBS1, HFBS2, HFBS3, HFBS4, HFBS5, HFBS6, HFBS7, and HFBS8, hydrolyzed faba bean starches. Assays were performed in triplicate. Mean ± SD, values in the same column with different superscript letters are significantly different (α = 0.05).

**Table 3:** Thermal parameters of native and hydrolyzed faba bean starches ^1^.

Sample	T_o_ (°C)	T_p_ (°C)	T_f_ (°C)	ΔH (J/g)
NFBS	62.5 ± 2.14 ^b^	68.0 ± 0.48 ^c^	73.8 ± 0.78 ^c^	10.9 ± 0.75 ^a^
CFBS	64.1 ± 0.17 ^b^	71.5 ± 0.45 ^b^	80.0 ± 0.19 ^b^	12.3 ± 0.77 ^a^
HFBS2	74.1 ± 1.12 ^a^	79.4 ± 1.86 ^a^	86.1 ± 0.95 ^a^	2.6 ± 1.28 ^b^
HFBS5	63.3 ± 0.07 ^b^	71.1 ± 0.11 ^bc^	80.4 ± 0.28 ^b^	11.0 ± 0.77 ^a^
HFBS8	65.4 ± 1.79 ^b^	70.7 ± 1.96 ^bc^	79.5 ± 0.54 ^b^	0.6 ± 0.11 ^b^

^1^ T_o_, onset temperature; T_p_, peak temperature; T_f_, final temperature; ΔH, gelatinization enthalpy. NFBS, native faba bean starch; CFBS, conventional hydrolysis faba bean starch; HFBS2, HFBS5, and HFBS8, hydrolyzed faba bean starches. Assays were performed in triplicate. Mean ± SD, values in the same column with different superscript letters are significantly different (α = 0.05).

**Table 4:** The 2^4−1^ fractional factorial design to explore starch acid hydrolysis by microwave energy.

Treatment Identification	Moisture, % (M)	Pure Hydrochloric Acid, mL/100 mL (A)	Time, s (T)	Power Level (P)
HFBS1	20	2	30	2
HFBS2	40	2	30	6
HFBS3	20	4	30	6
HFBS4	40	4	30	2
HFBS5	20	2	60	6
HFBS6	40	2	60	2
HFBS7	20	4	60	2
HFBS8	40	4	60	6

## Data Availability

Not applicable.

## Sample Availability

Samples of the compounds are available from the authors.
